# Maternal education and age: inequalities in neonatal death

**DOI:** 10.11606/S1518-8787.2017051007013

**Published:** 2017-11-07

**Authors:** Sandra Costa Fonseca, Patricia Viana Guimarães Flores, Kenneth Rochel Camargo, Rejane Sobrino Pinheiro, Claudia Medina Coeli

**Affiliations:** I Departamento de Epidemiologia e Bioestatística . Instituto de Saúde Coletiva . Universidade Federal Fluminense . Niterói , RJ , Brasil; II Programa de Pós-Graduação em Saúde Coletiva. Instituto de Estudos em Saúde Coletiva . Universidade Federal do Rio de Janeiro . Rio de Janeiro , RJ , Brasil; III Instituto de Medicina Social . Universidade do Estado do Rio de Janeiro . Rio de Janeiro , RJ , Brasil; IV Instituto de Estudos em Saúde Coletiva . Universidade Federal do Rio de Janeiro . Rio de Janeiro , RJ , Brasil

**Keywords:** Infant Mortality, trends, Socioeconomic Factors, Mothers, Educational Status, Maternal Age, Health Inequalities, Mortalidade Infantil, tendências, Fatores Socioeconômicos, Mães, Escolaridade, Idade Materna, Desigualdades em Saúde

## Abstract

**OBJECTIVE:**

Evaluate the interaction between maternal age and education level in neonatal mortality, as well as investigate the temporal evolution of neonatal mortality in each stratum formed by the combination of these two risk factors.

**METHODS:**

A nonconcurrent cohort study, resulting from a probabilistic relationship between the Mortality Information System and the Live Birth Information System. To investigate the risk of neonatal death we performed a logistic regression, with an *odds ratio* estimate for the combined variable of maternal education and age, as well as the evaluation of additive and multiplicative interaction. The neonatal mortality rate time series, according to maternal education and age, was estimated by the Joinpoint Regression program.

**RESULTS:**

The neonatal mortality rate in the period was 8.09‰ and it was higher in newborns of mothers with low education levels: 12.7‰ (adolescent mothers) and 12.4‰ (mother 35 years old or older). Low level of education, without the age effect, increased the chance of neonatal death by 25% (OR = 1.25, 95%CI 1.14–1.36). The isolated effect of age on neonatal death was higher for adolescent mothers (OR = 1.39, 95%CI 1.33–1.46) than for mothers aged ≥ 35 years (OR = 1.16, 95%CI 1.09–1.23). In the time-trend analysis, no age group of women with low education levels presented a reduction in the neonatal mortality rate for the period, as opposed to women with intermediate or high levels of education, where the reduction was significant, around 4% annually.

**CONCLUSIONS:**

Two more vulnerable groups – adolescents with low levels of education and older women with low levels of education – were identified in relation to the risk of neonatal death and inequality in reducing the mortality rate.

## RESUMO

**OBJETIVO:**

Avaliar a interação entre idade e escolaridade materna na mortalidade neonatal, assim como investigar a evolução temporal da mortalidade neonatal em cada estrato formado pela combinação desses dois fatores de risco.

**MÉTODOS:**

Estudo de coorte não concorrente, resultante de relacionamento probabilístico entre o Sistema de Informações sobre Mortalidade e o Sistema de Informações sobre Nascidos vivos. Para investigar o risco de óbito neonatal, foi realizada regressão logística, com estimativa de *odds ratio* para variável combinada escolaridade e idade materna, além da avaliação de interação aditiva e multiplicativa. A série temporal da taxa de mortalidade neonatal, de acordo com escolaridade e idade maternas, foi estimada pelo programa *Joinpoint regression*.

**RESULTADOS:**

A taxa de mortalidade neonatal no período foi de 8,09‰, sendo mais elevada em recém-nascidos de mães com baixa escolaridade: 12,7‰ (mães adolescentes) e 12,4‰ (mães com 35 anos e mais). A baixa escolaridade, sem efeito da idade, aumentou a chance de óbito neonatal em 25% (OR = 1,25; IC95% 1,14–1,36). Já o efeito isolado da idade no óbito neonatal foi maior para mães adolescentes (OR = 1,39; IC95% 1,33–1,46) do que para mães com idade ≥ 35 anos (OR = 1,16; IC95%1,09–1,23). Na análise temporal, nenhuma faixa etária de mulheres com baixa escolaridade apresentou redução da taxa de mortalidade neonatal no período, em contraponto às mulheres com escolaridade intermediária ou elevada, onde a redução foi significante, em torno de 4% anualmente.

**CONCLUSÕES:**

Detectaram-se dois grupos mais vulneráveis – adolescentes de baixa escolaridade e mulheres mais velhas de baixa escolaridade – em relação ao risco de óbito neonatal e à desigualdade na redução da taxa de mortalidade.

## INTRODUCTION

Social disparities in relation to infant and neonatal mortality have been investigated in public health^7^ ^,^ ^10^ ^,^ ^19^ ^,^ ^23^. Among the social determinants studied, maternal education level is one of the most corroborated. The ability to acquire knowledge on health issues and the optimal use of health services is attributed to high educational level^7^.

In several countries, including Brazil, there has been a progression in educational level in the last few decades for women, accompanied by an improvement in infant indicators^7^. In Brazil, progress in child health indicators has led the country to achieve, before the deadline, the Millennium Development Goal of reducing mortality of children under five years old by 2/3^21^.

Besides the improvement in maternal education, the reduction of inequalities in the country^15^ and interventions such as complementary sources of income and expansion of primary care^11^ contributed to the achievement of this goal. In the context of the public policies directed to children’s health, several programs were implemented in the last 20 years. The priority was the reduction of infant mortality through the organization of perinatal and newborn care and the humanization of prenatal care and birth^21^.

Despite the overall reduction in infant mortality, one of the challenges for Brazil is the reduction of neonatal mortality, which accounts for 2/3 of infant mortality and which had been decreasing at a much slower rate than that of post-neonatal mortality^21^ ^,^ ^23^.

One of the proposed explanations for the persistence of inequalities is that, as the educational level of the population increases, the group of women with low levels of education is selected, with a concentration of risk factors^10^. Another sociodemographic factor related to social disparities in perinatal health is maternal age, both for the group of women over 35 years old^14^ and for the adolescent girls^8^.

Our hypothesis is that an interaction between low education levels and extreme maternal ages would increase the risk of neonatal mortality above what would be expected by the isolated effect of each of these factors. Additionally, our interest is the time trend of the disparities, since few studies have evaluated this trend in low and middle-income countries^16^ ^,^ ^22^.

This study aimed to evaluate the interaction between maternal age and education in neonatal mortality, as well as to investigate the time trend of neonatal mortality in each stratum formed by the combination of these two risk factors, in a retrospective cohort of births.

## METHODS

A nonconcurrent cohort study which used a linked database of birth and neonatal death records of a population of live-born children of women residing in the state of Rio de Janeiro between 2004 and 2010. We chose this period due to the quality of the data, mainly the possibility of relating the bases and the comparative perspective with the policies implemented by the Stork Network in 2011^a^.

The integration of the databases of the identified live births records, the Live Birth Information System – SINASC (n = 1,519,095) and the neonatal mortality records from the Mortality Information System – SIM (n = 15,540 deaths with less than 28 days of life) from the period between 2004 and 2010 was performed using the method of probabilistic linkage of records^3^, using the program RecLink^3^. With this technique, we linked the information of 14,827 of the 15,540 neonatal deaths recorded in the SIM, with a sensitivity of the linkage procedure above 95%.

For inclusion in the study’s population, the registry should belong to a single-pregnancy live birth, the mother had to reside in the state of Rio de Janeiro at the time of delivery, with birth weight equal to or greater than 500 g and estimated gestational age equal to or greater than 22 weeks. Multiple deliveries were excluded because of the probability of false positive pairs occurrence in the probabilistic linkage. Records with ignored information were about 2% of the total.

### Statistical Analyses

Neonatal mortality rates were estimated along with their respective confidence intervals calculated by the exact method. Following the recommendations by Knol and Vanderweele^13^, a composite exposure variable was created, with six levels combining maternal age and education. The odds ratios (OR) of each level of the composite variable were presented using the group of 20–34-year-olds with education ≥ 4 years as reference^13^. We estimated the OR for neonatal mortality through the logistic regression model, using the Stata software (version 9). The additive interaction measures were calculated in an electronic spreadsheet developed by Andersson et al.^1^, which was powered with estimates of coefficients and covariance matrix. Three measures were calculated: the relative excess risk due to interaction (RERI), the proportion attributable to the interaction (AP), and the synergy index (S), as well as the respective confidence intervals estimated by the delta method^1^. These measures are calculated according to the following formulas: RERI = OR_11_ - OR_10_ - OR_01_ + 1; AP = RERI/OR_11_; S = [OR_11_ - 1] / [(OR_10_ - 1) + (OR_01_ - 1)]; where 0 in the subscript index indicates the reference category and 1 indicates the exposure category. When RERI = 0, AP = 0 and S = 1, no interaction is indicated.

Next, the effect of educational level in each age group was estimated, as well as the effect of age at each educational level, using logistic models. Finally, the multiplicative interaction was evaluated by means of a logistic model in which each variable was included separately (age and education) and a term of interaction between them was also included.

The time trend of the neonatal mortality rate (NMR) according to the same combined maternal age groups and education was described. This time trend analysis was performed through a joinpoint regression analysis, using the software Joinpoint Regression Program^b^, testing annual trends. The annual percentage change (APC) is estimated and considered significant when the curve differs from zero using the Monte Carlo Permutation method.

In the analysis, the category of low education levels was valued, with a cut-off point in four years compared to the others. It is understood that an education period lower than four years characterizes functional illiteracy and that this level impacts human development^c^.

Regarding the ethical aspects, the confidentiality of the data was guaranteed and the project was approved by the Ethics Committee of the Instituto de Estudos de Saúde Coletiva of the Universidade Federal do Rio de Janeiro (Declaration 69965, dated 8 August, 2012 – CAE 05373812.1.0000.5286).

## RESULTS

Throughout the period (2004–2010), 1,445,342 newborns were studied, of which 11,694 evolved to neonatal death (NMR = 8.09, 95%CI 7.94–8.24).

In the analysis of the joint distribution of maternal education and age (Table 1), the most vulnerable groups are evident. For neonatal mortality, both adolescents and older women with low educational levels had an NMR greater than 12 deaths per thousand live births, close to twice the NMR of the 20–34 age group.

**Table 1:** Distribution of neonatal mortality (per thousand live births) according to maternal age and education. State of Rio de Janeiro, 2004–2010.

Variable	Education	
Maternal age (years)	Low	Intermediate and high
	(< 4 years)	(≥ 4 years)
	(n = 91,973)	(n = 1,353,369)

	NMR (95%CI)	NMR (95%CI)
11–19	12.71 (10.94–14.67)	10.13 (9.74–10.52)
20–34	9.07 (8.35–9.83)	7.29 (7.12–7.46)
35 or older	12.47 (10.68–14.46)	8.44 (7.97–8.92)

NMR: neonatal mortality rate

In all age strata, children of mothers with less than four years of education presented a greater chance of neonatal death, when compared to the children of mothers with at least four years of education (Table 2, in bold). In the last two columns of Table 2, the OR related to the association between maternal age and the chance of neonatal mortality in each stratum of maternal education are presented. Children of adolescents and women aged 35 years old and older presented a higher chance of mortality when compared to mothers aged between 20 and 34 years, in both strata of maternal education.

**Table 2:** Odds ratios for neonatal mortality according to maternal age and education. State of Rio de Janeiro, 2004–2010.

Variable	Maternal age group (years)						OR (95%CI) for maternal age (10–19 years), within the maternal education strata	OR (95%CI) for maternal age (≥ 35 years), within the maternal education strata

	20-34		10-19		≥ 35			
		
	# of deaths/ survivors	OR (95%CI)	# of deaths/ survivors	OR (95%CI)	# of deaths/ survivors	OR (95%CI)		
Maternal education								
≥ 4 years	6,927/943,409	*Reference*	*2,601/254,274*	*1.39 (1.33–1.46)*	*1,233/144,925*	*1.16 (1.09–1.23)*	1.39 (1.33–1.46)	1.16 (1.09–1.23)
				*p = 0.000*		*p = 0.000*	p = 0.000	p = 0.000
< 4 years	578/63,161	*1.25 (1.14–1.36)*	*183/14,257*	*1.74 (1.51–2.03)*	*172/13,622*	*1.72 (1.48–2.00)*	1.40 (1.19–1.66);	1.38 (1.16–1.64)
		*p = 0.000*		*p = 0.000*		*p = 0.000*	p = 0.000	p = 0.000

OR (95%CI) for education within the maternal age strata								

		**1.25 (1.14–1.36)**		**1.25 (1.08–1.46)**		**1.48 (1.26–1.74)**		
		**p = 0.000**		**p = 0.003**		**p = 0.000**		
Interaction measure on the additive scale								
RERI (95%CI)			0.11 (-0.17–0.39); p = 0.45		0.32 (0.10–0.53); p = 0.003			
AP (95%CI)			0.06 (-0.09–0.21); p = 0.42		0.18 (0.06–0.31); p = 0.004			
S (95%CI)			1.17 (0.79–1.72); p = 043		1.78 (1.05–3.00);p = 0.032			
Interaction measure on the multiplicative scale OR (95%CI)			1.01 (0.85–1.20); p = 0.94		1.19 (0.99–1.43); p = 0.06			

OR: odds ratio; RERI: relative excess risk due to interaction; AP: proportion attributable to interaction; S: synergy index

The values in bold indicate the comparison between education levels for each age group.

The values in italics indicate the joint distribution between maternal age and education.

Also in Table 2 (in italics), the OR related to the analysis of the joint distribution between maternal age and education are presented. Low level of education, without the age effect, increased the chance of neonatal death by 25% (OR = 1.25, 95%CI 1.14–1.36). The isolated effect of age on neonatal death was higher for adolescent mothers (OR = 1.39, 95%CI 1.33–1.46) than for mothers aged ≥ 35 years (OR = 1.16, 95%CI 1.09–1.23). Children of mothers in the extremes of age and with low education levels presented approximately 1.7 times greater chance of evolving to neonatal death, when compared to the children of mothers between 20–34 years old and with education ≥ 4 years. However, only for the group of mothers aged ≥ 35 years old, there was a significant interaction between age and education in the occurrence of neonatal death, both in the additive and on the multiplicative scale, although in the latter the statistical significance was borderline (p = 0.06).

Table 3 shows the time trend of neonatal mortality in both periods. In adolescents with low education levels, an increase in NMR was observed, although it was not statistically significant. For women of low education levels in other age groups, the downward trend did not reach significance. On the other hand, all women with intermediate or high education levels had a significant reduction in NMR, regardless of age.

**Table 3:** Time trends of neonatal mortality rate (‰ live births) according to combined variable of maternal age and education. State of Rio de Janeiro, 2004–2010.

Maternal category/Education and age	Neonatal mortality rate							Annual percentage change
	
	2004	2005	2006	2007	2008	2009	2010	(95%CI)
Low education								
(1) 11 to 19 years	16.0	11.9	6.8	14.4	10.9	10.2	18.9	2.3% (-10.1–16.3)
(2) 20 to 34 years	10.3	8.7	8.2	9.5	8.8	8.9	7.7	-2.4% (-5.8–1.1)
(3) ≥ 35 years	13.3	16.0	10.2	10.1	13.1	12.9	9.5	-3.8 % (-10.5–3.4)
Intermediate or high education								
(4) 11 to 19 years	11.3	11.2	10.8	9.4	9.3	9.5	9.1	-4.0% (-5.8– -2.2)^*^
(5) 20 to 34 years	8.1	8.1	7.5	7.3	6.7	6.7	6.6	-4.1% (-4.9– -3.2)^*^
(6) ≥ 35 years	10.1	9.3	8.4	8.8	7.9	7.4	7.3	-5.2% (-6.3– -4.0)^*^

^*^ p < 0.05

## DISCUSSION

Despite the drop in neonatal mortality at the national and global levels^21^ ^,^ ^23^, our study identified that changes in neonatal mortality in the state of Rio de Janeiro were accompanied by social inequality, showing a reduction only for women with intermediate and higher education. Vulnerable groups were formed^5^, combining extremes of age and low education levels.

Although the combined effect of maternal age and low education levels has increased the odds of neonatal death by about 70% at both extremes of maternal age, the significant interaction between maternal age and low education levels was only observed among mothers aged ≥ 35 years old. One possible explanation for this finding is the greater magnitude of the lowest maternal age category on neonatal mortality.

Our results suggest that the simultaneous presence of these two risk factors characterizes population groups that, over the course of their lives, are exposed to multiple risk factors and to the development of various health problems^d^. Population interventions generally do not reach such groups, which may increase social disparities in health^6^.

Other studies in Brazil show disparate results. Hernandez et al. found an improvement in the educational level and reduction of inequalities in infant mortality in Porto Alegre, state of Rio Grande do Sul, from 1996 to 2008^12^. Garcia and Santana, using data from the National Household Sample Survey (PNAD) from 1993 to 2008, observed a reduction in inequalities for infant deaths^9^. Sousa et al. showed that the reduction in infant mortality in Brazil was at the expense of a decrease of the indicator in the richest groups, with persistent iniquities^19^. According to the authors^19^, for neonatal mortality between 1991 and 2000, the total reduction in the highest income quintile was 49%, while in the poorest quintile it was reduced only by 24%.

McKinnon et al. studied, through absolute and relative indicators of inequality, the reduction of the neonatal mortality rate (NMR) in low and middle-income countries^16^. The average reduction in NMR was associated with changes in indicators of educational inequality – moderate for absolute reduction and weak for relative reduction. Victora and Barros commented on these results, questioning which interventions in the health area were most effective for each type of reduction^22^. In countries where the NMR is not very high, the challenge is to make the most sophisticated interventions – which are technology dependent – accessible to all^20^ ^,^ ^22^. This may be the case in several more developed regions of Brazil, such as the one in this study.

When analyzing two variables usually investigated as distal factors of neonatal death^24^ – maternal age and education – we corroborate its association with neonatal death. As a limitation of this study, the non-inclusion of the skin color variable, still limited in the information systems, especially in SIM^4^, may have prevented a better understanding of the interrelationships between these sociodemographic factors^2^. However, based on other studies that analyzed the same variables, we believe that, even with adjustment for skin color, age and education would continue to have magnitude and statistical significance^17^.

On the other hand, one of the strengths of this study is the use of population data of universal scope (considering the coverage of SIM and SINASC in the country), which avoid selection bias present in adherence-dependent cohorts at follow-up or restricted to care in the Brazilian Unified Health System (SUS).

Future studies should deepen the understanding of the effects of disparities in relation to the proximal determinants (low birth weight, prematurity, and fetal growth) and intermediary determinants, such as differences in access to health services and maternal habits, such as smoking.

Also important in the future will be the analysis of the period from 2011 to 2015, after implementation of the Stork Network, compared with the period analyzed in this study, to evaluate the changes resulting from this strategy in maternal and child care.

It is worth emphasizing that population strategies without a due focus on the most vulnerable groups can paradoxically increase the disparities; on the other hand, the combination of intersectoral activities (e.g., in the field of education) and the social participation of the population itself are crucial in achieving the objectives^18^.

Although our work lacks the scope to identify the best strategy, we believe that, among different policy approaches^6^, broadening access to effective health services for defined vulnerable social groups is a viable health policy and should be on the agenda of governments, in the perspective of women and their babies.

## Footnotes

a Ministério da Saúde (BR). Portaria Nº 1.459, de 24 de junho de 2011. Institui, no âmbito do Sistema Único de Saúde - SUS - a Rede Cegonha. Brasília (DF); 2011 [citado 2017 Apr 29]. Available from: http://bvsms.saude.gov.br/bvs/saudelegis/gm/2011/prt1459_24_06_2011.html

b National Cancer Institute (US). Joinpoint Regression Program, Version 3 – April 2011: Statistical Methodology and Applications Branch and Data Modeling Branch, Surveillance Research Program. Available from: https://surveillance.cancer.gov/joinpoint/

c Instituto Brasileiro de Geografia e Estatística. Taxa de analfabetismo funcional. Rio de Janeiro: IBGE; 2001-2009 [cited 2017 Apr 29]. Available from: http://seriesestatisticas.ibge.gov.br/series.aspx?t=taxa-analfabetismo&vcodigo=PD384

d Milstein B. Hygeia’s constellation: navigating health futures in a dynamic and democratic world [dissertation]. Cincinatti: Graduate College of Interdisciplinary Arts and Sciences, Union Institute & University; 2006. Available from: http://www.uic.edu/sph/phtpg/Content/Reading%20Room/Articles/Hygeia’s_constellation_Milstein.pdf
